# Causes of Death in Patients with Severe Aortic Stenosis: An Observational study

**DOI:** 10.1038/s41598-017-15316-6

**Published:** 2017-11-07

**Authors:** Eri Minamino-Muta, Takao Kato, Takeshi Morimoto, Tomohiko Taniguchi, Hiroki Shiomi, Kenji Nakatsuma, Shinichi Shirai, Kenji Ando, Norio Kanamori, Koichiro Murata, Takeshi Kitai, Yuichi Kawase, Makoto Miyake, Chisato Izumi, Hirokazu Mitsuoka, Masashi Kato, Yutaka Hirano, Shintaro Matsuda, Kazuya Nagao, Tsukasa Inada, Tomoyuki Murakami, Yasuyo Takeuchi, Keiichiro Yamane, Mamoru Toyofuku, Mitsuru Ishii, Moriaki Inoko, Tomoyuki Ikeda, Akihiro Komasa, Eiji Tada, Katsuhisa Ishii, Kozo Hotta, Nobuya Higashitani, Toshikazu Jinnai, Yoshihiro Kato, Yasutaka Inuzuka, Chiyo Maeda, Yuko Morikami, Naritatsu Saito, Ryuzo Sakata, Kenji Minatoya, Takeshi Kimura

**Affiliations:** 1 Department of Cardiovascular Medicine, Kyoto University Graduate School of Medicine, Kyoto, Japan; 2 Department of Clinical Epidemiology, Hyogo College of Medicine, Nishinomiya, Japan; 3 Department of Cardiology, Kokura Memorial Hospital, Kokura, Japan; 4 Division of Cardiology, Shimada Municipal Hospital, Shimada, Japan; 5 Department of Cardiology, Shizuoka City Shizuoka Hospital, Shizuoka, Japan; 6 Department of Cardiovascular Medicine, Kobe City Medical Center General Hospital, Kobe, Japan; 7 Department of Cardiovascular Medicine, Kurashiki Central Hospital, Kurashiki, Japan; 8 Department of Cardiology, Tenri Hospital, Tenri, Japan; 9 Division of Cardiology, Nara Hospital, Kinki University Faculty of Medicine, Ikoma, Japan; 10 Department of Cardiology, Mitsubishi Kyoto Hospital, Kyoto, Japan; 11 Department of Cardiology, Kinki University Hospital, Osakasayama, Japan; 12 Department of Cardiovascular Center, Osaka Red Cross Hospital, Osaka, Japan; 13 Department of Cardiology, Koto Memorial Hospital, Higashiomi, Japan; 14 Department of Cardiology, Shizuoka General Hospital, Shizuoka, Japan; 15 Department of Cardiology, Nishikobe Medical Center, Kobe, Japan; 16 Department of Cardiology, Japanese Red Cross Wakayama Medical Center, Wakayama, Japan; 17 Department of Cardiology, National Hospital Organization Kyoto Medical Center, Kyoto, Japan; 18 Cardiovascular Center, The Tazuke Kofukai Medical Research Institute, Kitano Hospital, Osaka, Japan; 19 Department of Cardiology, Hikone Municipal Hospital, Hikone, Japan; 20 Department of Cardiology, Kansai Electric Power Hospital, Osaka, Japan; 21 Department of Cardiology, Hyogo Prefectural Amagasaki General Medical Center, Amagasaki, Japan; 22 Department of Cardiology, Japanese Red Cross Otsu Hospital, Otsu, Japan; 23 Department of Cardiology, Saiseikai Noe Hospital, Osaka, Japan; 24 Department of Cardiology, Shiga Medical Center for Adults, Moriyama, Japan; 25 Department of Cardiology, Hamamatsu Rosai Hospital, Hamamatsu, Japan; 26 Department of Cardiology, Hirakata Kohsai Hospital, Hirakata, Japan; 27 Department of Cardiovascular Surgery, Kobe City Medical Center General Hospital, Kobe, Japan; 28 Department of Cardiovascular Surgery, Kyoto University Graduate School of Medicine, Kyoto, Japan

## Abstract

Whether patients with severe aortic stenosis (AS) die because of AS-related causes is an important issue for the management of these patients. We used data from CURRENT AS registry, a Japanese multicenter registry, to assess the causes of death in severe AS patients and to identify the factors associated with non-cardiac mortality. We enrolled 3815 consecutive patients with a median follow-up of 1176 days; the 1449 overall deaths comprised 802 (55.3%) from cardiac and 647 (44.7%) from non-cardiac causes. Heart failure (HF) (25.7%) and sudden death (13.0%) caused the most cardiac deaths, whereas infection (13.0%) and malignancy (11.1%) were the main non-cardiac causes. According to treatment strategies, infection was the most common cause of non-cardiac death, followed by malignancy, in both the initial aortic valve replacement (AVR) cohort (N = 1197), and the conservative management cohort (N = 2618). Both non-cardiac factors (age, male, body mass index <22, diabetes, prior history of stroke, dialysis, anemia, and malignancy) and cardiac factors (atrial fibrillation, ejection fraction <68%, and the initial AVR strategy) were associated with non-cardiac death. These findings highlight the importance of close monitoring of non-cardiac comorbidities, as well as HF and sudden death, to improve the mortality rate of severe AS patients.

## Introduction

Aortic stenosis (AS) is the most common valvular disease worldwide. AS predominantly affects the elderly because age-related degenerative changes are the main etiologies. Older patients with AS have more comorbidities^1^. In patients with AS who undergo surgical or transcatheter interventions, detailed causes of deaths have previously been reported^2–6^. In a systematic review, non-cardiac deaths accounted for approximately half of the mortalities beyond 30 days after transcatheter aortic valve implantation (TAVI)^6^. In the PARTNER trial cohort B, the non-cardiovascular mortality rate was 43% at 2 years^5^. Causes of death are often analyzed in selected cohorts, such as candidates for surgical aortic valve replacement, or in randomized controlled studies wherein the relatively strict enrollment criteria limit participation. Although the natural course of AS is well known, there is limited literature on the exact causes of death in the population of patients with severe AS that was managed conservatively in the contemporary era^2,7^. High risk surgical candidates no longer undergo classical surgery, but receive TAVI, as do inoperable patients^8^. Theoretically, these patients are more likely to die because of non-cardiac causes.

Recently, we reported a multicenter observational registry characterized by the enrollment of all consecutive patients who met the criteria of severe AS in real clinical practice^9,10^. The present study aimed to investigate the cardiac and non-cardiac causes of death in patients with severe AS and to assess the associated factors with non-cardiac deaths in this cohort. The investigation of the causes of death can provide knowledge crucial to answer the question if the patients with AS die from AS-related causes or not, and how we should manage the patients in the era in which TAVI is becoming widely used.

## Methods

The CURRENT AS (Contemporary outcomes after sURgery and medical tREatmeNT in patients with severe Aortic Stenosis) registry enrolled 3815 consecutive patients with severe AS from 27 centers (on-site surgical facilities in 20 centers) in Japan between January 2003 and December 2011 (Appendix material)^9^. We searched the hospital database for transthoracic echocardiography and enrolled consecutive patients who met the definition of severe AS on their index echocardiography (peak aortic jet velocity [Vmax] >4.0 m/s, mean aortic pressure gradient [PG] >40 mm Hg, or aortic valve area [AVA] <1.0 cm^2^) for the first time during the study period. The study design and patient enrollment in the registry were previously described in detail^9^. Briefly, among the 3815 study patients, 1197 patients were managed with the initial aortic valve replacement (AVR) strategy, while 2618 patients were managed with the conservative strategy. In the present analysis, we (1) evaluated the cumulative incidence of all-cause, cardiac, and non-cardiac death during follow-up in the entire study population, (2) analyzed detailed causes of mortality according to the initial treatment strategies, and (3) determined the factors associated with non-cardiac mortality.

The study protocol was approved by the institutional review board of each participating center and was carried out in accordance with the approved guidelines. Given the retrospective nature of the study, the requirement of written informed consent was waived. All patients agreed to participate in the study when contacted for follow-up. The patient records/information was anonymized prior to analysis.

The CURRENT AS clinical events committee reviewed the documentation concerning every death that occurred after enrollment. The cause of death was classified according to the VARC (Valve Academic Research Consortium) definitions and adjudicated by a clinical event committee^11^. Sudden death was defined as unexplained death in a previously stable patient. Every death was placed into one of the 2 categories in the CURRENT AS registry: (1) cardiovascular deaths, which consist of heart failure (HF), aortic valve procedure death, myocardial infarction, sudden death, infective endocarditis, stroke, renal failure, aortic/peripheral vascular disease, other cardiac cause, and unknown death; and (2) non-cardiovascular deaths, which include malignancy, infection, respiratory failure, liver failure, renal failure, bleeding, trauma, and others^9^. In the present study, in order to distinguish cardiac causes from extra-cardiac causes, we used cardiac death and non-cardiac death instead of cardiovascular death and non-cardiovascular death, with deaths due to stroke and peripheral artery disease categorized as non-cardiac death because the thromboembolic events could result from the atherosclerotic burden. The classification of the causes of death is presented in Supplementary Table 1. Anemia was defined according to the World Health Organization criteria (hemoglobin <12.0 g/dL in women and <13.0 g/dL in men). The results of the two-dimensional transthoracic echocardiography were analyzed at baseline. The left ventricular ejection fraction (LVEF) was measured using the Teichholz method or the modified Simpson’s rule method.

### Statistical analysis

Categorical variables were expressed as numbers and percentages. Continuous variables were expressed as means (SD) or medians with interquartile range (IQR).

The cumulative incidences of all-cause, cardiac, and non-cardiac death were assessed by the Kaplan-Meier method. Causes of deaths in the conservative management and initial AVR cohorts were presented according to the intention-to-treat principle regardless of whether AVR was actually performed. We developed a cause-specific Cox proportional hazard model for non-cardiac death, and assessed the factors associated with non-cardiac death. Consistent with our previous study^9^, we included 22 clinically relevant variables listed in Supplementary Table 2 and the treatment strategy at baseline in the model for non-cardiac death, and calculated the adjusted hazard ratios (HRs) and 95% confidence intervals (CIs).

All statistical analyses were conducted by two physicians (E.M. and T.K.) and a statistician (T.M.) using JMP 10.0.2 (SAS Institute Inc., Cary, North Carolina). All P values were 2-tailed, and the level of statistical significance was set at P < 0.05.

## Results

### Baseline characteristics

The mean age of the current study population was 77.8 ± 9.8 years, and 1443 (38%) patients were male. At baseline, there were 2005 symptomatic patients (53%), and 1808 asymptomatic patients (47%), excluding 2 patients without information on symptoms. Of the study population, 70% had hypertension, 24% had diabetes mellitus, 15% had end-stage renal disease, and 55% had anemia. A history of malignancy was documented in 14% of patients, and 3.9% of patients were currently under treatment for malignancy. The median Society of Thoracic Surgeons (STS) score was 3.8 (IQR: 2.2–16.7). The mean LVEF was 63 ± 14% and the mean Vmax was 4.1 ± 0.9 m/s (Supplementary Table 2).

### Incidence and causes of death during follow-up

**Figure 1:**
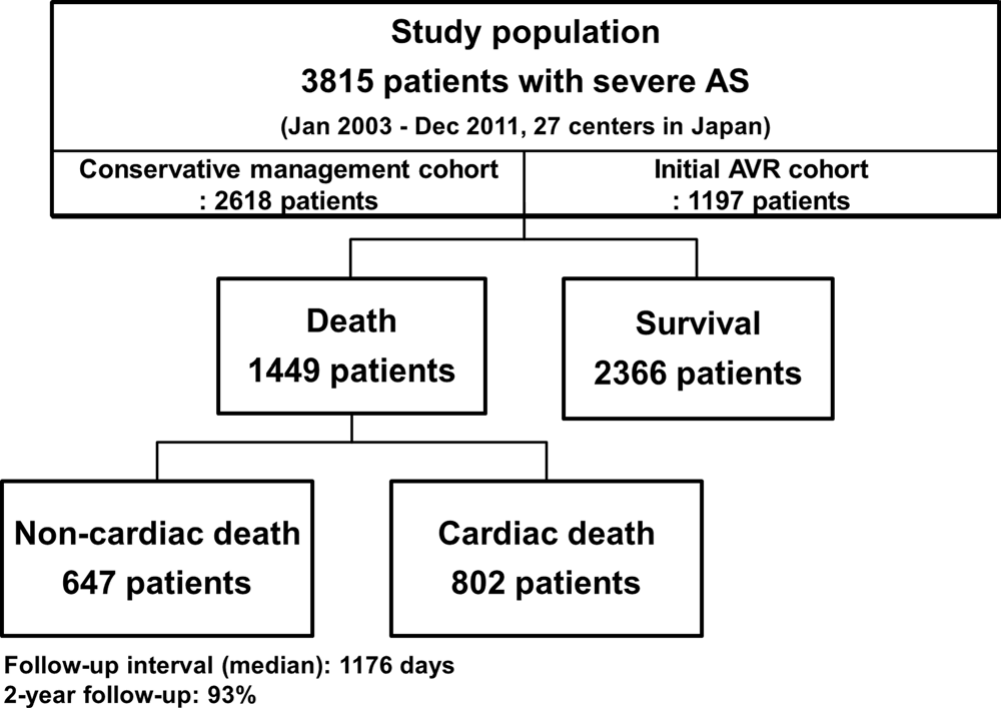
Study patient flow. AVR = aortic valve replacement, AS = aortic stenosis.

**Figure 2:**
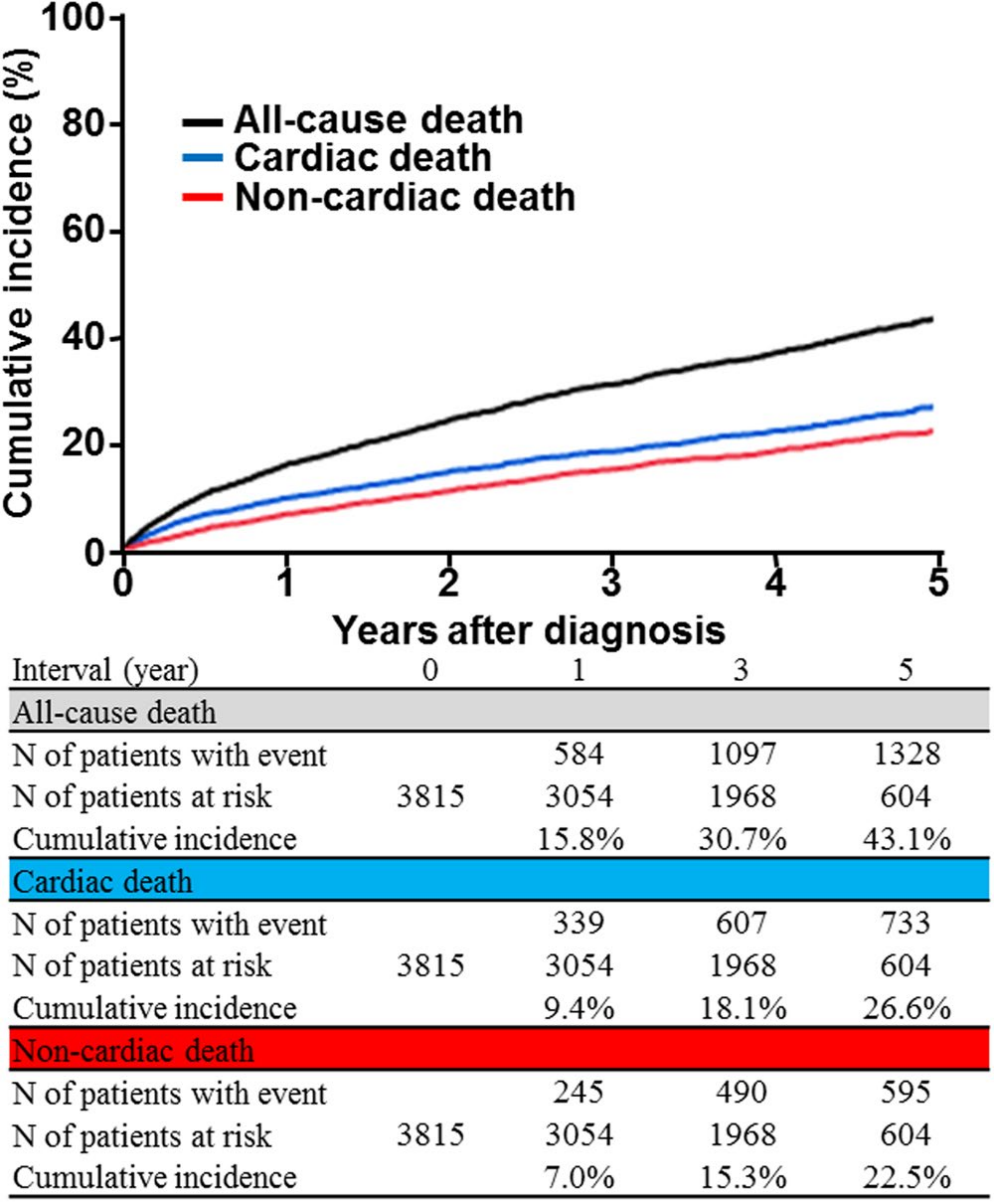
Cumulative incidence of all-cause, cardiac, and non-cardiac death.

**Figure 3:**
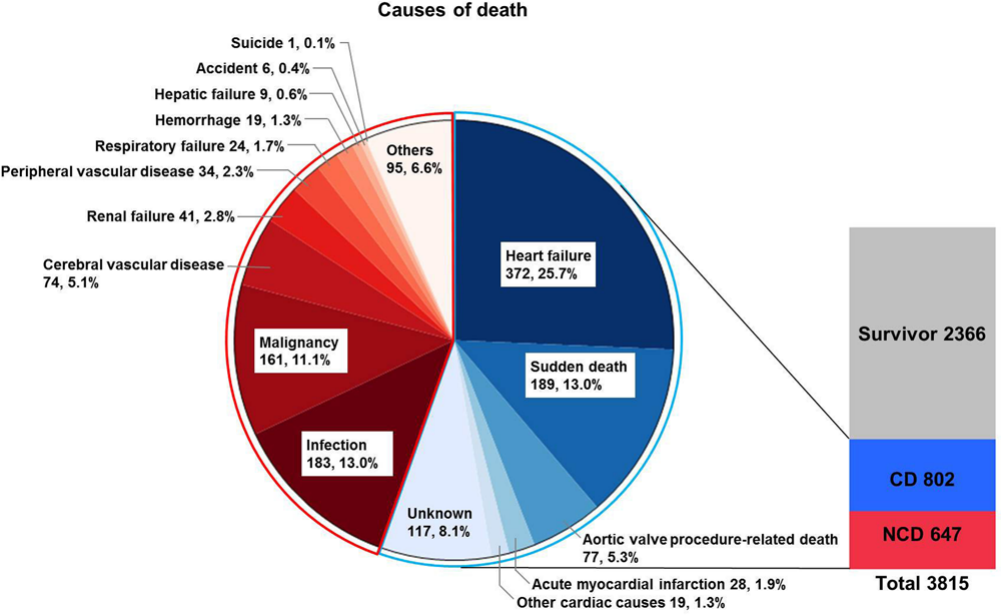
Causes of death. CD = cardiac death, and NCD = non-cardiac death.

The median follow-up period was 1176 (IQR: 733–1618) days, with a 93% follow-up rate at 2 years. Of the 1449 observed deaths, 802 deaths (55.3%) were cardiac and 647 deaths (44.7%) were non-cardiac (Fig. 1). The Kaplan-Meier curves showed a constant increase in cardiac death as well as non-cardiac death, with cumulative 5-year incidences of 43.1%, 26.6%, and 22.5% for all-cause, cardiac, and non-cardiac death, respectively (Fig. 2). HF and sudden death were the two leading causes of cardiac death, followed by procedure (AVR/TAVI)-related death (Fig. 3). Infection and malignancy were the two leading causes of non-cardiac death, followed by cerebrovascular death and renal failure (Fig. 3).

### Causes of death according to management strategy

Regarding the initial treatment strategies, 2618 patients were managed conservatively, while 1197 patients were managed with the initial AVR strategy. In the conservative management cohort, there were 674 cardiac deaths and 539 non-cardiac deaths. In the initial AVR cohort, there were 128 cardiac deaths and 108 non-cardiac deaths. Procedure-related death was the leading cause of cardiac death in the initial AVR cohort, followed by HF and sudden death. In the conservative management cohort, HF was the leading cause of cardiac death, followed by sudden death. Infection was the most common cause of non-cardiac death, followed by malignancy, in both cohorts (Supplementary Figure 1).

### The factors independently associated with non-cardiac deaths

The factors independently associated with non-cardiac deaths are provided in Table 1. Age, male sex, body mass index <22, diabetes in insulin therapy, prior symptomatic stroke, prior history of atrial fibrillation, dialysis, anemia, malignancy currently under treatment, LVEF below 68%, and the initial AVR strategy were associated with non-cardiac mortality (Table 1).

**Table 1:** Cause-specific Cox proportional hazard models for factors associated with non-cardiac death.

Variable	Non-cardiac death N = 647		
	N of patients with event (proportion among patients with event)	Adjusted HR (95% CI)	P value
Age		1.08 (1.07–1.09)	<0.001
Male	281 (43%)	1.53 (1.28–1.82)	<0.001
BMI <22^||^	467 (72%)	1.33 (1.11–1.60)	0.002
Hypertension	457 (71%)	0.86 (0.72–1.03)	0.09
Current smoking	26 (4.0%)	0.92 (0.60–1.35)	0.69
Diabetes mellitus on insulin therapy	46 (7.1%)	1.55 (1.12–2.10)	0.01
Prior symptomatic stroke	135 (21%)	1.38 (1.13–1.67)	0.002
Prior myocardial infarction	62 (9.6%)	0.89 (0.65–1.20)	0.45
Atrial fibrillation	181 (28%)	1.36 (1.13–1.63)	0.001
Aortic/peripheral vascular disease	66 (10%)	1.32 (1.00–1.72)	0.053
Serum creatinine >0.83	386 (60%)	1.11 (0.92–1.33)	0.27
Dialysis	105 (16%)	2.85 (2.21–3.65)	<0.001
Anemia^§^	451 (70%)	1.48 (1.23–1.78)	<0.001
Liver cirrhosis	13 (2.0%)	1.69 (0.91–2.88)	0.09
Malignancy currently under treatment	65 (10%)	3.19 (2.41–4.14)	<0.001
Lung disease ≥moderate	27 (4.2%)	1.33 (0.88–1.92)	0.18
Coronary artery disease	195 (30%)	0.98 (0.80–1.20)	0.87
Vmax ≥4 m/s	304 (47%)	0.94 (0.79–1.11)	0.44
LVEF <68%	448 (69%)	1.41 (1.19–1.68)	<0.001
Any valvular disease	288 (45%)	1.02 (0.86–1.21)	0.80
TR pressure gradient >40 mmHg	107 (17%)	0.99 (0.79–1.23)	0.93
Admission for heart failure at Index echocardiography	144 (22%)	1.07 (0.87–1.31)	0.49
Initial AVR cohort	108 (17%)	0.58 (0.46–0.73)	<0.001

AVR = aortic valve replacement, BMI = body mass index, CI = confidence interval, and HR = hazard ratio, LVEF = left ventricular ejection fraction, TR = tricuspid regurgitation, and Vmax = peak aortic jet velocity.

^||^Body mass index was calculated as weight in kilograms divided by height in meters squared.

^§^Anemia was defined by the World Health Organization criteria (hemoglobin <12.0 g/dL in women and <13.0 g/dL in men).

## Discussion

The main findings of this study are as follows: (1) HF and sudden death are the top two causes of cardiac death whereas infection and malignancy are the top two causes of non-cardiac death in patients with severe AS; and (2) according to the results of the cause-specific analysis, LVEF <68%, atrial fibrillation, and the initial AVR strategy along with age, male sex, body mass index <22, and comorbidities, are the factors associated with non-cardiac death.

The impact of non-cardiac death was not negligible in patients with severe AS, although cardiac death was the main contributor to the overall mortality. In patients who were treated surgically, the most common cause of cardiac death was procedure-related death, reflecting the perioperative risk in the current patients with severe AS, followed by HF as the second most frequent cause of death. As anticipated, patients with severe AS who were managed conservatively were more likely to die of cardiac causes, such as HF and sudden death. A certain proportion of this conservatively-managed cohort is thought to be candidates of TAVI, but also those with disabilities prohibitive for surgery or TAVI, and those who refused surgery^9,12^. The main non-cardiac causes of death in the entire population and in each strategy were infection and malignancy. These findings highlight the importance of paying close attention to the non-cardiac comorbidities. In patients who were managed surgically, the risk of cardiac death was substantially decreased after surgery^1,4,6^, increasing the impact of non-cardiac death afterwards. Given that TAVI, which is widely employed in intermediate- to high-risk patients with severe AS^13^, decreases mortality and positively affects symptoms and functional status, non-cardiac death can contribute much more to the overall mortality in patients who undergo TAVI.

We used cause-specific Cox hazard models to determine the factors associated with non-cardiac death, because we focused on the etiologies of non-cardiac deaths^14,15^. This study revealed that the multiple factors related to non-cardiac deaths can be divided into the two large categories: non-cardiac factors (comorbidities, age, male sex, anemia, etc.) and cardiac factors. Non-cardiac factors were tightly linked to non-cardiac deaths. Prior stroke, diabetes on insulin therapy, and dialysis might indicate the increased atherosclerotic burden^16–18^; anemia suggested possible iron deficiency, chronic inflammation, and malignancy^19^. Furthermore, low LVEF was related to non-cardiac death. Poor LV function is suggested to cause cardiac cachexia^20^ and may affect systemic immunity^21,22^, which leads to non-cardiac deaths such as infection and malignancy. Atrial fibrillation is associated with an increased incidence of cerebrovascular death^23^. The initial AVR strategy was associated with a lower risk of non-cardiac death, for which there might be three possible reasons. First, there is the possibility that AVR improved the systemic condition through improvement of the cardiac status. Second, patients managed with the initial AVR strategy might have been more closely followed-up at the outpatient department. Third, patients managed with the initial AVR strategy did not include those with disabilities prohibitive for surgery and those who refused surgery because of their limited life expectancy, which might not be adequately adjusted by the measured confounders. Although a cause–effect relationship between these above factors and non-cardiac deaths could not be demonstrated in the present study, cardiac factors may be linked to non-cardiac deaths and would be the possible therapeutic targets in patients with severe AS.

### Limitations

This study has several limitations. First, we did not show a statistical difference in the causes of death, because comprehensive presentation of mortality data was the main purpose of this study. Categorization of the circumstances surrounding each death, particularly the mechanism of death, were related to the process of adjudication and may be incomplete. Second, the present results in Japan cannot be easily generalized to reflect those of other countries and the world population. Third, this is a retrospective, observational, and epidemiological investigation mainly from the pre-TAVI era. Finally, there remains unmeasured confounders affecting the mortality in the multivariable models exploring the risk factors associated with non-cardiac death.

## Conclusions

Among cardiac deaths in severe AS patients (55.3%), HF and sudden death were the two main causes in the entire and conservative management cohorts. Procedure-related death was the leading cause of cardiac death in the initial AVR cohort. In addition, death from non-cardiac causes (44.7%), including death from infection and malignancy, is an important contributor to mortality in patients with severe AS.

## Electronic supplementary material

Supplementary material
