# Supplementary material for: Causes of Death in Patients with Severe Aortic Stenosis: An Observational study
Source: Sci Rep. 2017 Nov 7;7:14723. doi: 10.1038/s41598-017-15316-6 (PMC5676690; doi:10.1038/s41598-017-15316-6)
Supplement: Supplementary file 1 — Supplementary material [file 41598_2017_15316_MOESM1_ESM.pdf]

Supplementary materials

Causes of Death in Patients with Severe Aortic Stenosis:

An Observational study

Authors: Eri Minamino-Muta, MD[1], Takao Kato, MD[1]; Takeshi Morimoto, MD, MPH[2]; Tomohiko Taniguchi, MD[1]; Hiroki Shiomi, MD[1]; Kenji Nakatsuma, MD[1]; Shinichi Shirai, MD[3]; Kenji Ando, MD[3]; Norio Kanamori, MD[4]; Koichiro Murata, MD[5]; Takeshi Kitai, MD[6]; Yuichi Kawase, MD[7]; Makoto Miyake, MD[8]; Chisato Izumi, MD[8]; Hirokazu Mitsuoka, MD[9]; Masashi Kato, MD[10]; Yutaka Hirano, MD[11]; Shintaro Matsuda, MD[1]; Kazuya Nagao, MD[12]; Tsukasa Inada, MD[12]; Tomoyuki Murakami, MD[13]; Yasuyo Takeuchi, MD[14]; Keiichiro Yamane, MD[15]; Mamoru Toyofuku, MD[16]; Mitsuru Ishii, MD[17]; Moriaki Inoko, MD[18]; Tomoyuki Ikeda, MD[19]; Akihiro Komasa, MD[1]; Eiji Tada, MD[20]; Katsuhisa Ishii, MD[20]; Kozo Hotta, MD[21]; Nobuya Higashitani, MD[22]; Toshikazu Jinnai, MD[22]; Yoshihiro Kato, MD[23]; Yasutaka Inuzuka, MD[24]; Chiyo Maeda, MD[25]; Yuko Morikami, MD[26]; Naritatsu Saito, MD[1]; Ryuzo Sakata, MD[27]; Kenji Minatoya, MD[28]; Takeshi Kimura, MD[1].

Supplementary material content

**Supplementary Tables**	**Page 3-7**
**Supplementary Figures**	**Page 8-9**
**List of investigators**	**Page 10-12**

Supplementary Tables

Supplementary Table 1. Classifications of causes of death into cardiac and non-cardiac categories

Supplementary Table 2. Baseline clinical and echocardiographic characteristics

Supplemental Table 1. Classifications of causes of death into cardiac and non-cardiac categories

Cardiac death

Any of the following criteria

- Death due to acute myocardial infarction, heart failure, and other cardiac cause (e.g. tamponade)
- All aortic valve procedure-related death, including directly related to the procedure or complications during hospitalization after AVR/TAVI
- Sudden or unwitnessed death
- Death of unknown cause in which cardiac cause could not be excluded

Non-cardiac death

- Death caused by non-coronary vascular disease such as cerebrovascular disease, peripheral arterial disease, dissecting aneurysm, or other vascular disease
- Other non-cardiac deaths were sub-classified into the following categories: malignancy, accidental, infection/sepsis, respiratory failure, renal failure, hepatic failure, or other (e.g. senility, hemorrhage, suicide)

AVR=aortic valve replacement, TAVI=transcatheter aortic valve implantation

Supplementary Table 2. Baseline clinical and echocardiographic characteristics

**Variable**	**All patients (N=3815)**
**Clinical characteristics**	
Age, mean (SD), years *	77.8 (9.8)
Age ≥80 years, No. (%)	1729 (45)
Male, No. (%) *	1443 (38)
BMI, mean (SD)	21.8 (3.8)
BMI <22, No. (%), || *	2326 (61)
BSA, mean (SD), m^2	1.46 (0.18)
Initial AVR cohort, No. (%)	1197 (31)
Hypertension, No. (%) *	2667 (70)
Current smoking, No. (%) *	196 (5.1)
History of smoking, No. (%)	830 (22)
Dyslipidemia, No. (%), †	1327 (35)
On statin therapy, No. (%)	970 (25)
Diabetes mellitus, No. (%)	897 (24)
On insulin therapy, No. (%) *	188 (4.9)
Coronary artery disease, No. (%) *	1144 (30)
Prior myocardial infarction, No. (%) *	323 (8.5)
Prior PCI, No. (%)	502 (13)
Prior CABG, No. (%)	199 (5.2)
Prior symptomatic stroke, No. (%) *	503 (13)
Atrial fibrillation or flutter, No. (%) *	828 (22)
Aortic/peripheral vascular disease, No. (%) *	282 (7.4)
Serum creatinine, median (IQR), mg/dL *	0.9 (0.7-1.26)
Dialysis *	405 (11)
Anemia, No. (%),§ *	2117 (55)
Hemoglobin (IQR), g/dl	11.6 (10.2-13)
Liver cirrhosis (Child–Pugh B or C), No. (%) *	38 (1.0)
Malignancy, No. (%)	517 (14)
Malignancy currently under treatment *	149 (3.9)
Recurrence of malignancy, No. (%)	58 (1.5)
Chest wall irradiation, No. (%)	25 (0.7)
Immunosuppressive therapy, No. (%)	131 (3.4)
Chronic lung disease, No. (%)	400 (10)
Chronic lung disease (moderate or severe) *	112 (2.9)

Logistic EuroSCORE, median (IQR), %	9.7 (5.8-16.7)
EuroSCORE II, median (IQR), %	2.9 (1.62-4.82)
STS score (PROM), median (IQR), %	3.8 (2.2-6.5)
Symptoms at index echocardiography, No. (%)	2005 (53)
Chest pain	498 (13)
Syncope	198 (5.2)
Chronic exertional dyspnea	1603 (42)
Admission for heart failure at index echocardiography, No. (%)*	790 (21)
Etiology of aortic stenosis, No. (%)	
Degenerative	3379 (89)
Congenital (Unicuspid, Bicuspid, or Quadricuspid)	258 (6.8)
Rheumatic	150 (3.9)
Infective endocarditis	7 (0.2)
Others	21 (0.6)
**Echocardiographic variables**	
Vmax, mean (SD), m/s	4.1 (0.9)
Vmax ≥4m/s, No. (%) *	2185 (57)
Peak aortic PG, mean (SD), mmHg	71.8 (31.6)
Mean aortic PG, mean (SD), mmHg	40.8 (19.7)
AVA (equation of continuity), mean (SD), cm^2	0.72 (0.18)
AVA index, mean (SD), cm^2/m^2	0.50 (0.13)
LV end-diastolic diameter, mean (SD), mm	45.9 (7.0)
LV end-systolic diameter, mean (SD), mm	30.2 (7.9)
LVEF, mean (SD), %	62.8 (13.5)
LVEF <68%, No. (%) *	2242 (59)
IVST in diastole, mean (SD), mm	11.3 (2.3)
PWT in diastole, mean (SD), mm	11.0 (2.0)
ny combined valvular disease (moderate or severe), No. (%) *	1558 (41)
Moderate or severe AR	791 (21)
Moderate or severe MS	133 (3.5)
Moderate or severe MR	763 (20)
Moderate or severe TR	628 (16)
TR pressure gradient ≥40 mmHg, No. (%) *	606 (16)

* Variables selected for cause-specific Cox proportional hazard models.

|| Body mass index was calculated as weight in kilograms divided by height in meters squared.

† Dyslipidemia was defined as total cholesterol levels ≥240 mg/dL, high-density lipoprotein

cholesterol levels <40 g/dL, or the use of statin
§ Anemia was defined by the World Health Organization criteria (hemoglobin <12.0 g/dL in women and <13.0 g/dL in men).
AR=aortic regurgitation, AVA=aortic valve area, AVR=aortic valve replacement, BMI=body mass index, BSA=body surface area, CABG=coronary artery bypass grafting, ESRD=end-stage renal disease, IQR=interquartile range, IVST=interventricular septal wall thickness, LV=left ventricular, LVEF=left ventricular ejection fraction, MR=mitral regurgitation, MS=mitral stenosis, PCI=percutaneous coronary intervention, PG=pressure gradient, PROM=predicted risk of mortality, PWT=posterior wall thickness, SD=standard deviation, and STS=Society of Thoracic Surgeons, TR=tricuspid regurgitation, and Vmax=peak aortic jet velocity

Supplementary figure legend

Supplementary Figure 1. Causes of death according to management strategy

AVR=aortic valve replacement, CD=cardiac death, and NCD=non-cardiac death

Supplementary Figure 1.

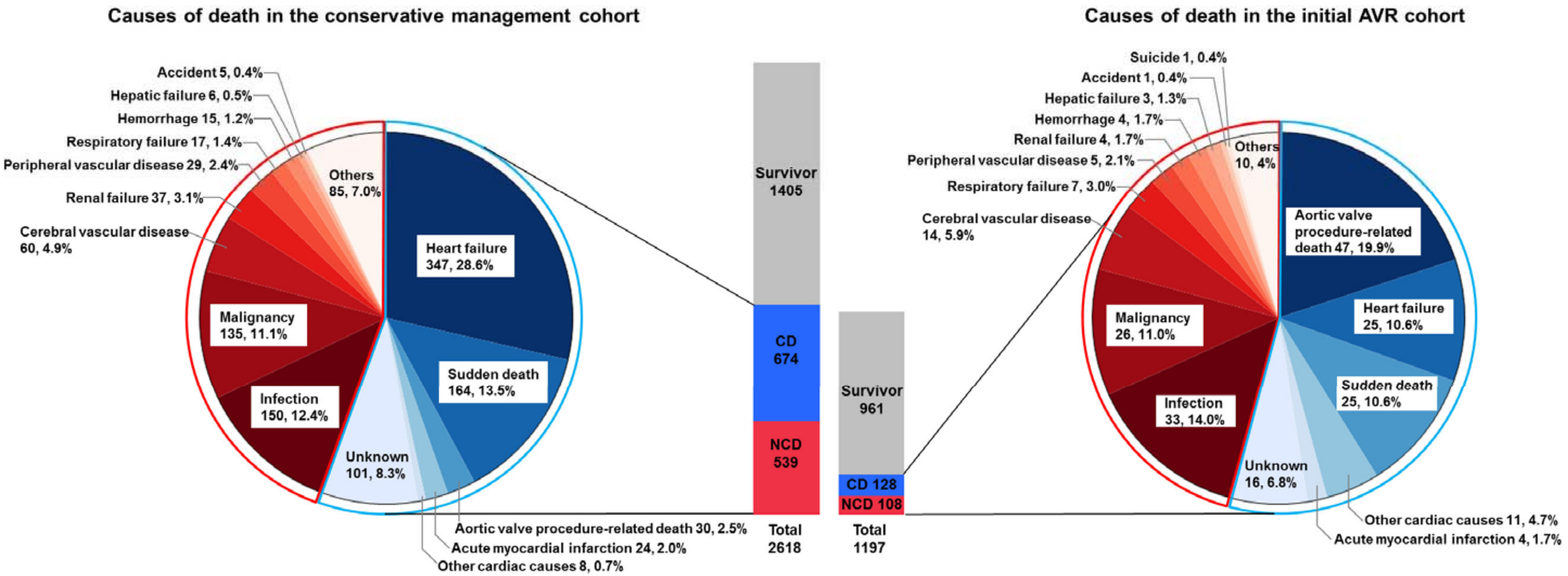

List of Investigators

Principal Investigators

Takeshi Kimura, Department of Cardiovascular Medicine, Kyoto University Graduate School of Medicine, Kyoto, Japan

Ryuzo Sakata, Department of Cardiovascular Surgery, Kyoto University Graduate School of Medicine, Kyoto, Japan

List of participating centers and investigators for the CURRENT AS registry

Cardiology

Department of Cardiovascular Medicine, Kyoto University Graduate School of Medicine: Takeshi Kimura, Tomohiko Taniguchi, Hiroki Shiomi, Naritatsu Saito, Masao Imai, Junichi Tazaki, Toshiaki Toyota, Hirooki Higami, Tetsuma Kawaji

Department of Cardiology, Kokura Memorial Hospital: Kenji Ando, Shinichi Shirai, Kengo Kourai, Takeshi Arita, Shiro Miura, Kyohei Yamaji

Division of Cardiology, Shimada Municipal Hospital: Takeshi Aoyama, Norio Kanamori

Department of Cardiology, Shizuoka City Shizuoka Hospital: Tomoya Onodera, Koichiro Murata

Department of Cardiovascular Medicine, Kobe City Medical Center General Hospital: Yutaka Furukawa, Takeshi Kitai, Kitae Kim

Department of Cardiovascular Medicine, Kurashiki Central Hospital: Kazushige Kadota, Yuichi Kawase, Keiichiro Iwasaki, Hiroshi Miyawaki, Ayumi Misao, Akimune Kuwayama, Masanobu Ohya, Takenobu Shimada, Hidewo Amano

Department of Cardiology, Tenri Hospital: Yoshihisa Nakagawa, Chisato Izumi, Makoto Miyake, Masashi Amano, Yusuke Takahashi, Yusuke Yoshikawa, Shunsuke Nishimura, Maiko Kuroda

Division of Cardiology, Nara Hospital, Kinki University Faculty of Medicine: Manabu Shirotani, Hirokazu Mitsuoka

Department of Cardiology, Mitsubishi Kyoto Hospital: Shinji Miki, Tetsu Mizoguchi, Masashi Kato, Takafumi Yokomatsu, Akihiro Kushiyama, Hidenori Yaku, Toshimitsu Watanabe

Department of Cardiology, Kinki University Hospital: Shunichi Miyazaki, Yutaka Hirano

Department of Cardiology, Kishiwada City Hospital: Mitsuo Matsuda, Shintaro Matsuda, Sachiko Sugioka

Department of Cardiovascular Center, Osaka Red Cross Hospital: Tsukasa Inada, Kazuya Nagao, Naoki Takahashi, Kohei Fukuchi

Department of Cardiology, Koto Memorial Hospital: Tomoyuki Murakami, Hiroshi Mabuchi, Teruki Takeda, Tomoko Sakaguchi, Keiko Maeda, Masayuki Yamaji, Motoyoshi

Maenaka, Yutaka Tadano
Department of Cardiology, Shizuoka General Hospital: Hiroki Sakamoto, Yasuyo Takeuchi, Makoto Motooka, Ryusuke Nishikawa
Department of Cardiology, Nishikobe Medical Center: Hiroshi Eizawa, Keiichiro Yamane, Mitsunori Kawato, Minako Kinoshita, Kenji Aida
Department of Cardiology, Japanese Red Cross Wakayama Medical Center: Takashi Tamura, Mamoru Toyofuku, Kousuke Takahashi, Euihong Ko
Department of Cardiology, National Hospital Organization Kyoto Medical Center: Masaharu Akao, Mitsuru Ishii, Nobutoyo Masunaga, Hisashi Ogawa, Moritake Iguchi, Takashi Unoki, Kensuke Takabayashi, Yasuhiro Hamatani, Yugo Yamashita
Cardiovascular Center, The Tazuke Kofukai Medical Research Institute, Kitano Hospital: Moriaki Inoko, Eri Minamino-Muta, Takao Kato
Department of Cardiology, Hikone Municipal Hospital: Yoshihiro Himura, Tomoyuki Ikeda
Department of Cardiology, Kansai Electric Power Hospital: Katsuhisa Ishii, Akihiro Komasa
Department of Cardiology, Hyogo Prefectural Amagasaki General Medical Center: Yukihito Sato, Kozo Hotta, Shuhei Tsuji
Department of Cardiology, Rakuwakai Otowa Hospital: Yuji Hiraoka, Nobuya Higashitani
Department of Cardiology, Saiseikai Noe Hospital: Ichiro Kouchi, Yoshihiro Kato
Department of Cardiology, Shiga Medical Center for Adults: Shigeru Ikeguchi, Yasutaka Inuzuka, Soji Nishio, Jyunya Seki
Department of Cardiology, Hamamatsu Rosai Hospital: Eiji Shinoda, Miho Yamada, Akira Kawamoto, Chiyo Maeda
Department of Cardiology, Japanese Red Cross Otsu Hospital: Takashi Konishi, Toshikazu Jinnai, Kouji Sogabe, Michiya Tachiiri, Yukiko Matsumura, Chihiro Ota
Department of Cardiology, Hirakata Kohsai Hospital: Shoji Kitaguchi, Yuko Morikami

Cardiovascular Surgery
Department of Cardiovascular Surgery, Kyoto University Graduate School of Medicine: Ryuzo Sakata, Kenji Minakata
Department of Cardiovascular Surgery, Kokura Memorial Hospital: Michiya Hanyu
Department of Cardiovascular Surgery, Shizuoka City Shizuoka Hospital: Fumio Yamazaki
Department of Cardiovascular Surgery, Kobe City Medical Center General Hospital: Tadaaki Koyama
Department of Cardiovascular Surgery, Kurashiki Central Hospital: Tatsuhiko Komiya
Department of Cardiovascular Surgery, Tenri Hospital: Kazuo Yamanaka

Department of Cardiovascular Surgery, Nara Hospital, Kinki University Faculty of Medicine: Noboru Nishiwaki
Department of Cardiovascular Surgery, Mitsubishi Kyoto Hospital: Hiroyuki Nakajima, Motoaki Ohnaka, Hiroaki Osada, Katsuaki Meshii
Department of Cardiovascular Surgery, Kinki University Hospital: Toshihiko Saga
Department of Cardiovascular Surgery, Kishiwada City Hospital: Masahiko Onoe
Department of Cardiovascular Surgery, Osaka Red Cross Hospital: Shogo Nakayama
Department of Cardiovascular Surgery, Shizuoka General Hospital: Genichi Sakaguchi
Department of Cardiovascular Surgery, Japanese Red Cross Wakayama Medical Center: Atsushi Iwakura
Department of Cardiovascular Surgery, National Hospital Organization Kyoto Medical Center: Kotaro Shiraga
Department of Cardiovascular Surgery, Cardiovascular Center, The Tazuke Kofukai Medical Research Institute, Kitano Hospital: Koji Ueyama
Department of Cardiovascular Surgery, Hyogo Prefectural Amagasaki General Medical Center: Keiichi Fujiwara
Department of Cardiovascular Surgery, Rakuwakai Otowa Hospital: Atsushi Fukumoto
Department of Cardiovascular Surgery, Shiga Medical Center for Adults: Senri Miwa
Department of Cardiovascular Surgery, Hamamatsu Rosai Hospital: Junichiro Nishizawa
Department of Cardiovascular Surgery, Japanese Red Cross Otsu Hospital: Mitsuru Kitano

A clinical event committee

Hirotoshi Watanabe, MD (Kyoto University Graduate School of Medicine); Kenji Nakatsuma, MD (Kyoto University Graduate School of Medicine), Tomoki Sasa, MD (Kishiwada City Hospital)
